# Supplementary material for: Influence of Block-Copolymers’ Composition as Compatibilizers for Epoxy/Silicone Blends
Source: Molecules. 2023 Aug 28;28(17):6300. doi: 10.3390/molecules28176300 (PMC10488889; doi:10.3390/molecules28176300)
Supplement: Supplementary file 1 [file molecules-28-06300-s001.zip › molecules-2502366-supplementary_Corrected Proofreading(3).pdf]

Influence of Block-Copolymers Composition As Compatibilizers for Epoxy/Silicone Blends

Christelle Delaite [1,2*], Sophie Bistac [1,2] and Daniela Rusu [1,2]

Supplementary Materials

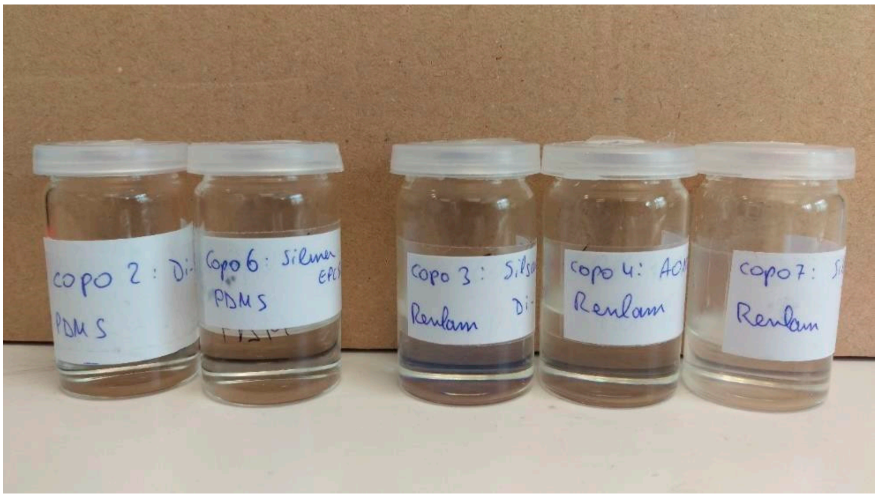

Figure S1. Fully miscible formulations.

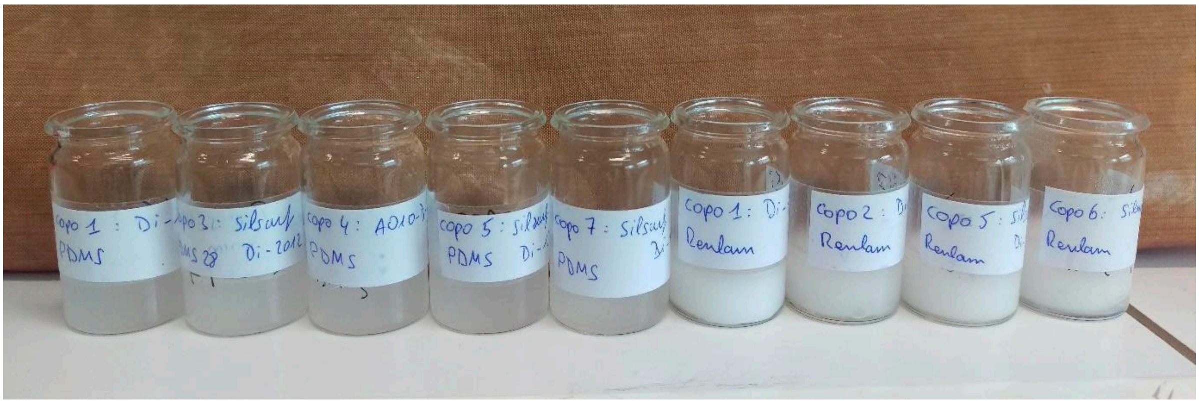

Figure S2. Formulations with partial miscibility (left) or immiscibility (right).

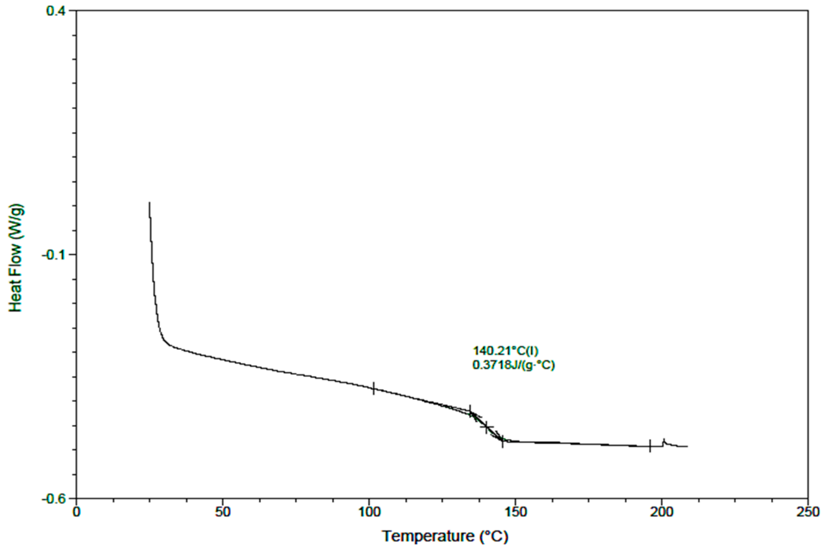

Figure S3. DSC curve of a neat DGEBA/IPD epoxy, after one hour of curing at 150°C (first ramp).

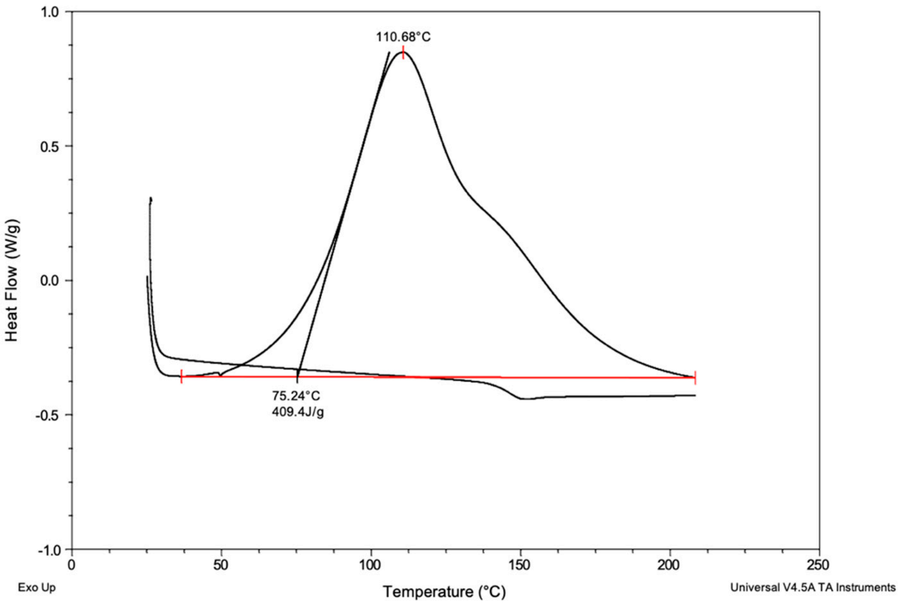

Figure S4. DSC curve of a liquid epoxy/silicone blend (5% PDMS) compatibilized with coPo3 (two ramps).

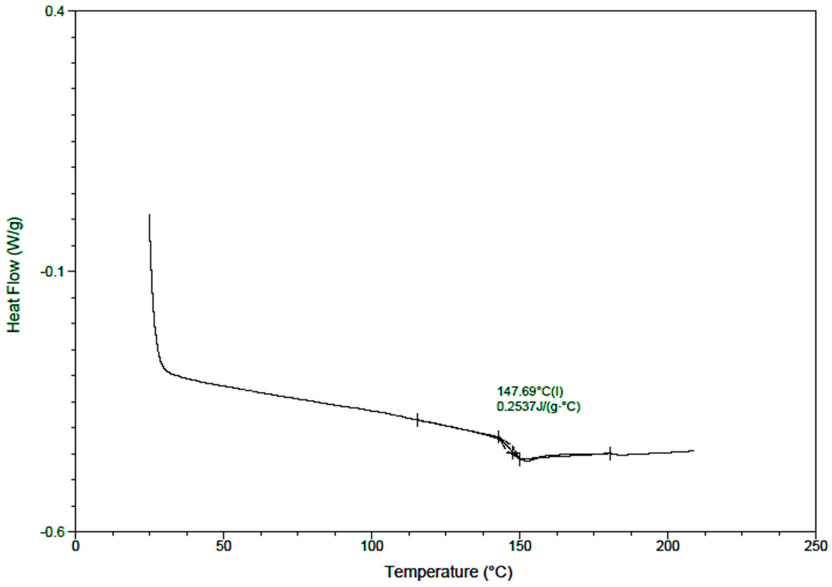

Figure S5. DSC curve of a cured epoxy/silicone blend (10% PDMS) compatibilized with coPo3 (first ramp).

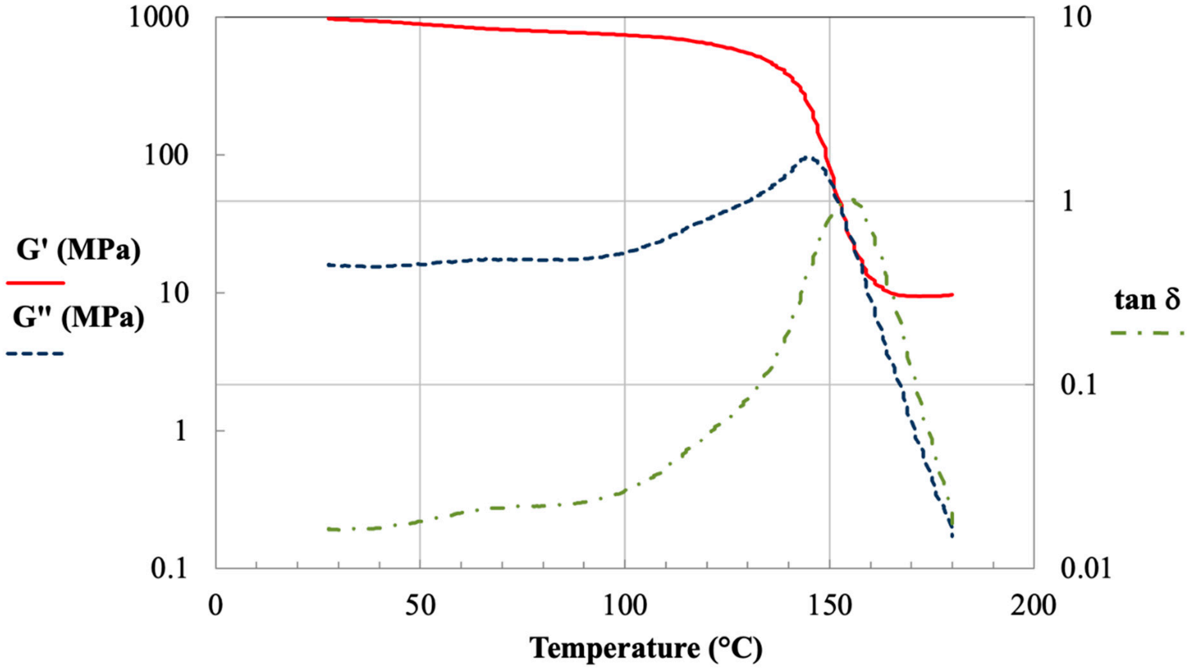

Figure S6. DMA of fully cured DGEBA/IPD neat epoxy.

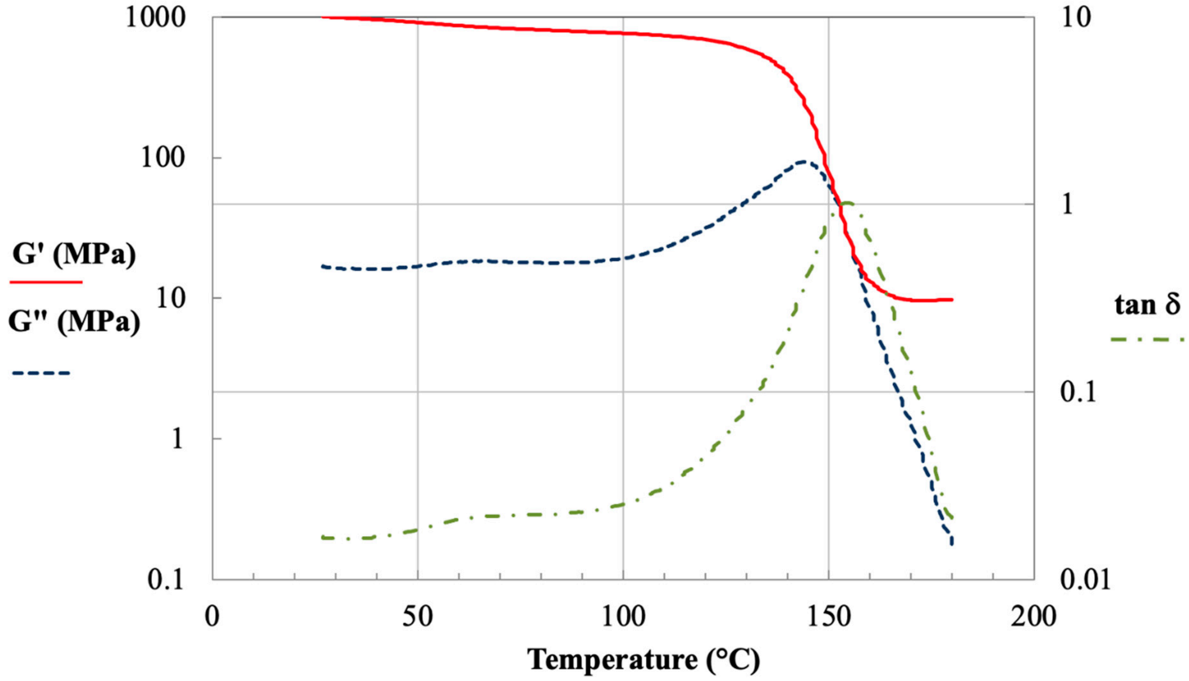

Figure S7. DMA of a fully cured epoxy/silicone blend (10% of PDMS) compatibilized with coPo3.
